# Investigation of Tongxie-Yaofang formula in treating ulcerative colitis based on network pharmacology via regulating MAPK/AKT signaling pathway

**DOI:** 10.18632/aging.205467

**Published:** 2024-01-24

**Authors:** Xinhong Liu, Mao Ye, Yinglin He, Qin Lai, Bo Liu, Leichang Zhang

**Affiliations:** 1 Department of Proctology, Affiliated Hospital of Jiangxi University of Traditional Chinese Medicine, Nanchang 330000, China; 2 Formula-Pattern Research Center of Traditional Chinese Medicine, Nanchang 330000, China

**Keywords:** Tongxie-Yaofang formula, ulcerative colitis, network pharmacology, SRC, MAPK

## Abstract

Background: Ulcerative colitis (UC) is a subtype of inflammatory bowel disease, which often leads to bloody diarrhea and abdominal pain. In this study, the function mechanism of Tongxie-Yaofang formula (TXYF) on UC was investigated.

Methods: Action targets of TXYF were obtained by Traditional Chinese Medicine Systems Pharmacology Database (TCMSP) and Traditional Chinese Medicine Integrated Database (TCMID) databases. The targets of UC were screened in Gene Cards and Online Mendelian Inheritance in Man (OMIM) databases. The network pharmacology of active ingredient targets was established via Cytoscape.

Results: A total of 42 chemical components and 5806 disease targets were obtained. The GO functional analysis showed that biological processes such as oxidative stress and molecular response to bacteria, molecular function such as protein and nucleic acid binding activity were significantly enriched. The top 20 KEGG enriched signal pathways indicated that the targets were mainly linked with IL-17, TNF, HIF-1. Molecular docking results showed that naringenin had good binding activity between naringin and MAPK, albiflorin and SRC. The activity of MPO, the concentration of HIF-1, IL-17 and TNF-α were significantly decreased after TXYF treatment. The characteristics of UC such as crypt distortion, crypt atrophy, and increased basal plasmacytosis were also less observed with the treatment of TXYF. What’s more, TXYF suppresses the phosphorylation of SRC, MAPK and AKT1 in UC.

Conclusions: TXYF showed treatment effect on UC through multiple components and multiple targets, which lays a foundation for further study of UC treatment.

## INTRODUCTION

Ulcerative colitis (UC) is a subtype of inflammatory bowel disease (IBD) of unknown etiology, which affects the colon and rectum, often leading to bloody diarrhea and abdominal pain [1, 2]. UC has a variety of pathogenesis, such as environmental factors, genetic susceptibility and intestinal microbiome dysfunction [3, 4]. In addition, UC patients may have unpredictable symptoms, such as fever, diarrhea, anemia, and intestinal perforation [5]. Although rectal Corticosteroid and biological drugs are the main methods to treat UC [6]. Nevertheless, with the increasing incidence rate and prevalence of UC worldwide, the government should understand the management and economic burden of this disease [7]. Therefore, it is urgent to seek more treatments with less side effects to treat UC [8, 9].

Traditional Chinese medicine (TCM) treatment has significant advantages over the simple use of Western medicine in improving patient symptoms, reducing intestinal mucosal permeability, improving intestinal inflammation, reducing recurrence rate, and regulating overall body function [10, 11]. Tongxie-Yaofang formula (TXYF) was first recorded in the “Danxi Heart Method” and is an effective formula for treating abdominal pain and diarrhea due to liver hyperactivity and spleen deficiency [12]. It has the effects of regulating the liver and spleen, relieving pain, and stopping diarrhea [13]. TXYF is composed of four kinds of TCM: *Atractylodes macrocephala*, *Radix Paeoniae Alba*, *Citrus sinensis (L.)*, and *Divaricate Saposhnikovia Root*. Modern medicine believes that TXYF can treat acute and chronic colitis [14, 15]. Meanwhile, the application of TXYF in other fields has been widely reported. TXYF regulated macrophage polarization to ameliorate DSS-induced colitis via NF-κB/NLRP3 signaling pathway [16]. Inhibitory effect of TXYF on colonic contraction in rats was reported [17]. TXYF shown good treatment effect on irritable bowel syndrome with diarrhea and type 2 diabetes mellitus in rats with liver-depression and spleen-deficiency [18]. However, systems biology research methods such as network pharmacology still need to be used to elucidate mechanisms of TXYF and provide scientific basis for expanding the scope of clinical applications in UC.

This study focuses on the effective active ingredients and targets of action of TXYF in treating UC by constructing a related network of “compounds of TXYF and targets of Disease”. Besides, the GO and KEGG functional enrichments were conducted to find out the biological process and underlying signaling pathways of TXYF in treating UC. Finally, the UC rats’ model was constructed to verify the protecting role of TXYF and its target signaling pathways in UC. This study lays a foundation for the utilization of TXYF in treatment for colitis.

## RESULTS

### Acquisition of active ingredients and targets of TXYF in UC

**Figure 1:**
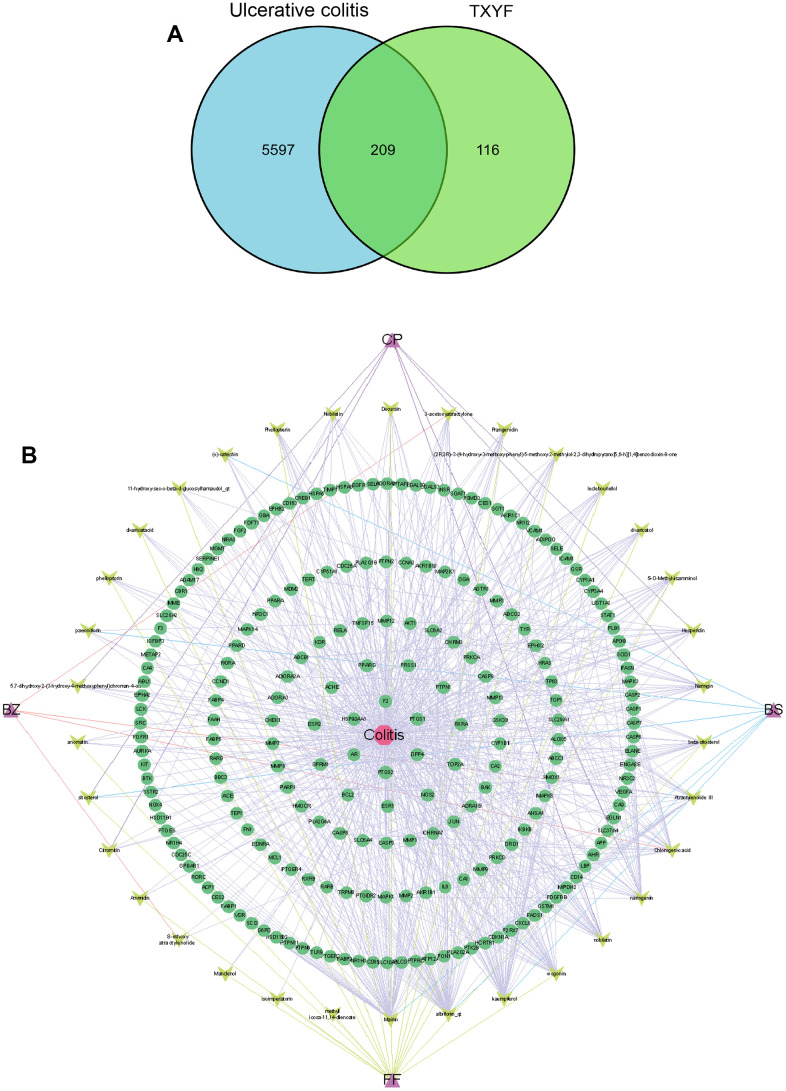
**The network of active ingredient-effective target of TXYF in UC.** (**A**) The Venn graph illustrated 209 common targets in-between 325 targets of TXYF and 5806 UC-related targets. (**B**) The network of active ingredient-effective target of TXYF in UC. Tongxie-Yaofang formula (TXYF).

Firstly, the 42 active ingredients in TXYF (Table 1) were obtained from TCMSP, TCMID database (screening criteria OB ≥ 30% and DL ≥ 0.18), and Pharmaceuticals (screening criteria: GI absorption=high, Druglikeness ≥ 2 yes). The total of 325 targets of Traditional Chinese Medicine was retrieved through TCMSP and TCMID database. Secondly, the UC-related genes were downloaded from Gene Card and OMIM databases, and targets with a score greater than the median are set as potential disease targets. Combining the relevant targets retrieved from the OMIM database, the duplicate values were deleted after merging, resulting in 5806 UC-related targets. Thirdly, the 209 common targets in-between 325 targets of TXYF and 5806 UC-related targets were obtained, which can be seen in Figure 1A.

**Table 1:** The active ingredients of Tongxie-Yaofang formula.

**ID**	**Molecule name**	**OB (%)**	**DL**
MOL001910	11alpha,12alpha-epoxy-3beta-23-dihydroxy-30-norolean-20-en-28,12beta-olide	64.773893	0.376
MOL001918	paeoniflorgenone	87.593121	0.367
MOL001919	(3S,5R,8R,9R,10S,14S)-3,17-dihydroxy-4,4,8,10,14-pentamethyl-2,3,5,6,7,9-hexahydro-1H-cyclopenta [a] phenanthrene-15,16-dione	43.556202	0.533
MOL001921	Lactiflorin	49.121317	0.797
MOL001924	paeoniflorin	53.870375	0.787
MOL001928	albiflorin_qt	66.640769	0.326
MOL001930	benzoyl paeoniflorin	31.274473	0.746
MOL000211	Mairin	55.377073	0.776
MOL000358	beta-sitosterol	36.913906	0.751
MOL000359	sitosterol	36.913906	0.751
MOL000422	kaempferol	41.88225	0.241
MOL000492	(+)-catechin	54.826434	0.242
MOL000020	12-senecioyl-2E, 8E, 10E-atractylentriol	62.396467	0.223
MOL000021	14-acetyl-12-senecioyl-2E, 8E, 10E-atractylentriol	60.312871	0.305
MOL000022	14-acetyl-12-senecioyl-2E, 8Z, 10E-atractylentriol	63.370918	0.3
MOL000028	α-Amyrin	39.51209	0.763
MOL000033	Cyclopenta [a] phenanthren-3-ol	36.228471	0.783
MOL000049	3β-acetoxyatractylone	54.066717	0.219
MOL000072	8β-ethoxy atractylenolide III	35.950919	0.211
MOL000359	sitosterol	36.913906	0.751
MOL004328	naringenin	59.293898	0.211
MOL005100	5,7-dihydroxy-2-(3-hydroxy-4 methoxyphenyl) chroman-4-one	47.736437	0.272
MOL005815	Citromitin	86.904047	0.514
MOL005828	nobiletin	61.669439	0.517
MOL000173	wogonin	30.684567	0.229
MOL000358	beta-sitosterol	36.913906	0.751
MOL001494	Mandenol	41.9962	0.193
MOL001942	isoimperatorin	45.464247	0.225
MOL003588	Prangenidin	36.314494	0.219
MOL007514	methyl icosa-11, 14-dienoate	39.667059	0.229
MOL013077	Decursin	39.267206	0.383

### The active-ingredient and effective-target network of TXYF in UC

Next, Cytoscape 3.7.2 was used to construct the active-ingredients and effective-target network of TXYF, as shown in Figure 1B. The topological parameters of the network for treating UC with TXYF were calculated to evaluate the importance of active ingredients and action targets. The results showed that active ingredients such as *quercetin*, *naringin*, *baicalin*, and *albiflorin* can act on multiple targets, which may be the main active ingredients of TXYF in treating UC.

### The construction of protein-protein interaction (PPI) network

**Figure 2:**
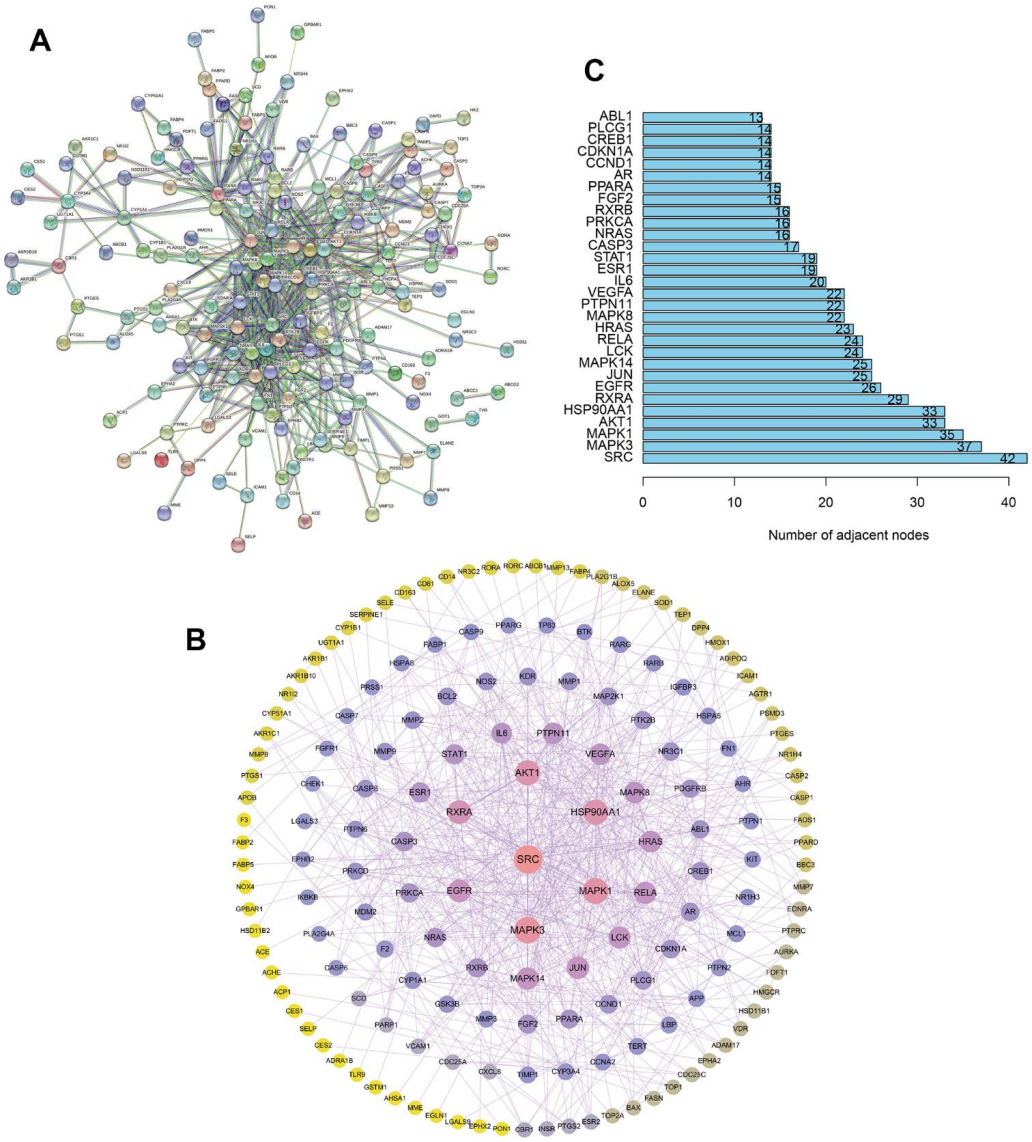
**The construction of protein-protein interaction network.** (**A**) The construction of interaction network based on the common targets via STRING database. (**B**) The construction of protein-protein interaction (PPI) network based on the common targets via Cytoscape software. (**C**) The number of adjacent nodes of the top 30 hub genes in PPI network.

Thereafter, the 209 common targets were uploaded to the STRING database to set the confidence level to be ≥ 0.9, and the interaction network diagram was obtained (Figure 2A). Then, the protein-protein interaction (PPI) network was constructed by Cytoscape (Figure 2B). The larger the node calculated in PPI network, the greater the degree value. According to Figure 2C, the targets at the center of the network include SRC, MAPK, AKT1, etc., were important targets for the treatment of UC with TXYF.

### The function and pathway enrichment analysis of targets

**Figure 3:**
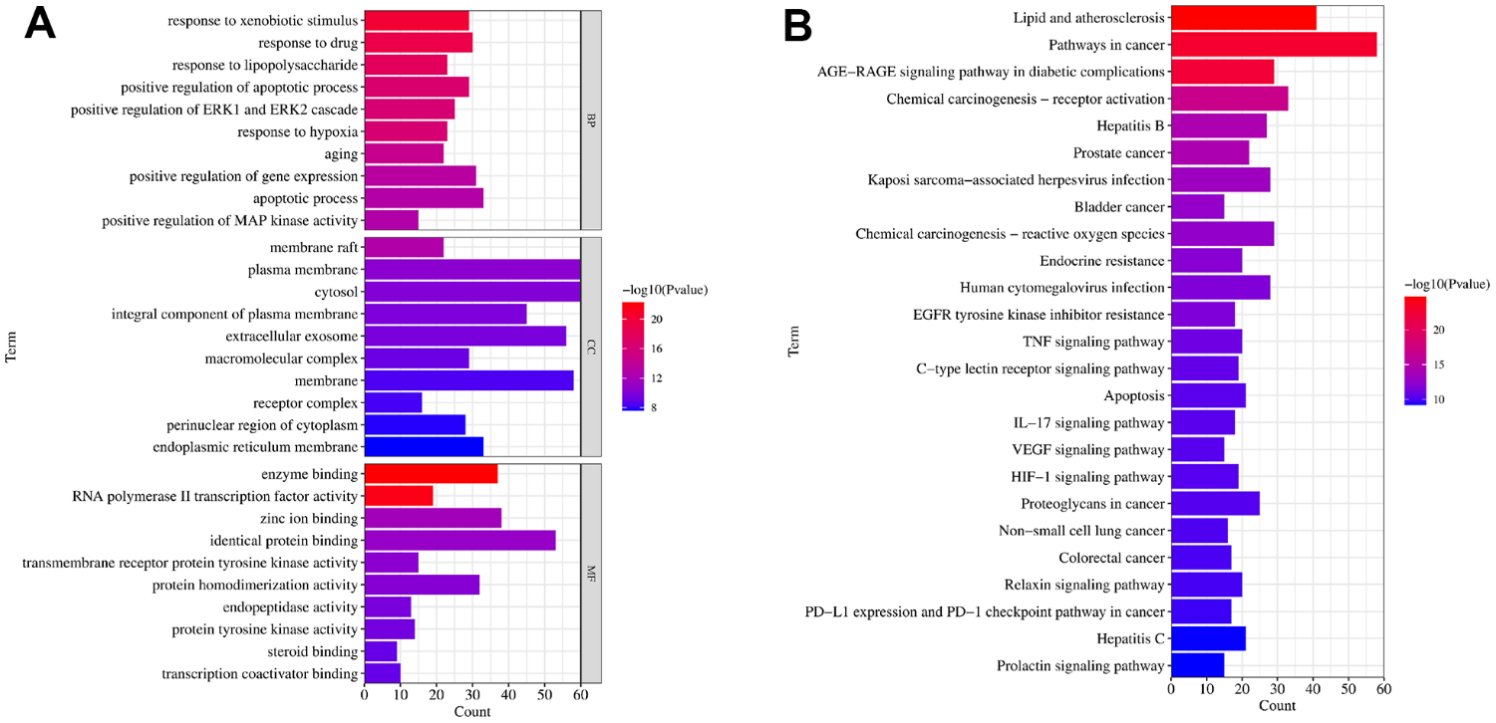
**The GO and KEGG functional enrichment analysis.** (**A**) The GO terms of biological process (BP), cellular compound (CC) and molecular function (MF) enrichment analysis of common targets. (**B**) The KEGG signaling pathways enrichment analysis of common targets. Gene Ontology (GO), Kyoto Encyclopedia of Genes and Genomes (KEGG).

In the next step, the aforementioned common targets were carried out for GO terms and KEGG enrichment analysis. As shown in Figure 3A, the targets were mainly involved in the biological processes like oxidative stress, molecule response to bacteria; The cellular compound was mainly related to membrane structure; and these targets were involved in molecular function such as protein and nucleic acid binding activity. In addition, the KEGG enrichment analysis identified 115 pathway signaling pathways, and the top 20 obtained from them indicated that they mainly play a role in UC with pathways such as AGE-RAGE, IL-17, TNF, HIF-1, etc. The top 20 pathways were selected for visualization, as shown in Figure 3B.

### Molecular docking of active ingredients in TXYF

**Figure 4:**
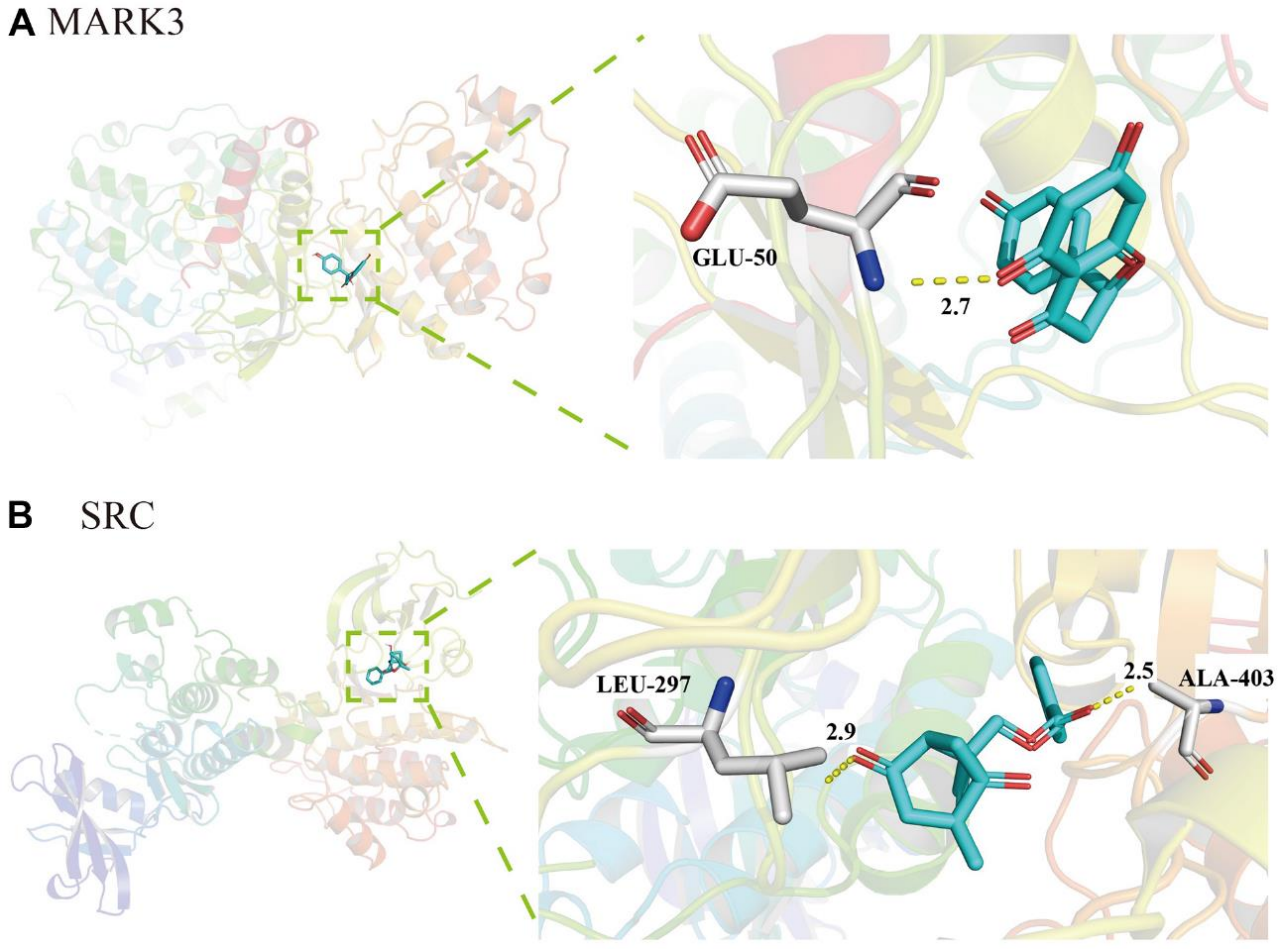
**Molecular docking of active ingredients in TXYF.** (**A**) The molecular docking diagram of *naringin* and MAPK. (**B**) The molecular docking diagram of albiflorin and SRC.

AutoDock Vina was used for molecular docking of active ingredients in TXYF. When the binding energy<-5.0 kcal/mol indicates a good binding activity between the two; When the binding energy<-7.0 kcal/mol indicates strong binding activity between the ligand and the receptor. As presented in Figure 4A, the binding energies of *naringin* and MAPK was -8.5 (kcal/mol), indicating strong binding activity between the drug and the target. Besides, there was also strong binding energies between *albiflorin* and SRC (-6.4 kcal/mol) (Figure 4B). These results suggest that the MAPK and SRC are vital target that TXYF active in UC.

### The protective effect of TXYF on UC rat model

**Figure 5:**
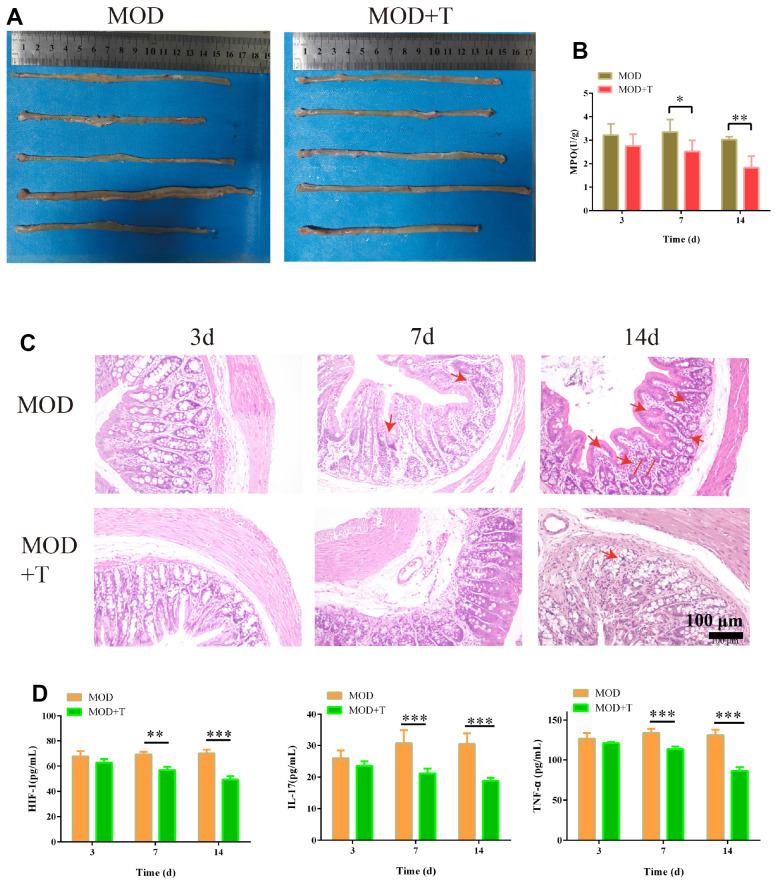
**The protective effect of TXYF on UC rat model.** (**A**) The colon in UC model group (MOD) and UC model+ TXYF group (MOD+T) at 14^th^ day after the UC model was established. (**B**) The activity of Myeloperoxidase (MPO) in MOD and MOD+T groups was measured at 3^rd^, 7^th^, and 14^th^ day after the UC model was established. (**C**) The representative images of hematoxylin-eosin (H&E) staining of colon collected from MOD and MOD+T groups at 3^rd^, 7^th^, and 14^th^ day after the UC model was established. (**D**) The concentration of HIF-1, IL-17 and TNF-α in MOD and MOD+T groups was measured at 3^rd^, 7^th^, and 14^th^ day after the UC model was established. N=5, *p<0.05, **p<0.01, and ***p<0.001.

In the following study, the UC rat model was established for further investigation. As shown in Figure 5A, shorter ulcerative intestinal tissue was observed in colon of UC group than UC + TXYF group at 14^th^ day. Besides, the activity of inflammation biomarker MPO [19] was gradually decreasing in UC + TXYF compared with UC group (Figure 5B). According to the HE staining of colon (Figure 5C), the characteristics of UC such as crypt distortion, crypt atrophy, and increased basal plasmacytosis were observed in UC group, while these characteristics were less appearance with the treatment of TXYF. IL-17, TNF, and HIF-1 signaling pathways were enriched as the potential signaling pathway of targets in the above results (Figure 3B). Interestingly, the concentration of inflammatory factors (HIF-1, IL-17 and TNF-α) were considerably decreased since the 7^th^ day with the treatment TXYF (p < 0.01) (Figure 5D). These results indicated that TXYF have protective effect on UC rat model by suppressing the inflammation.

### TXYF suppresses the phosphorylation of SRC, MAPK and AKT1 in UC

**Figure 6:**
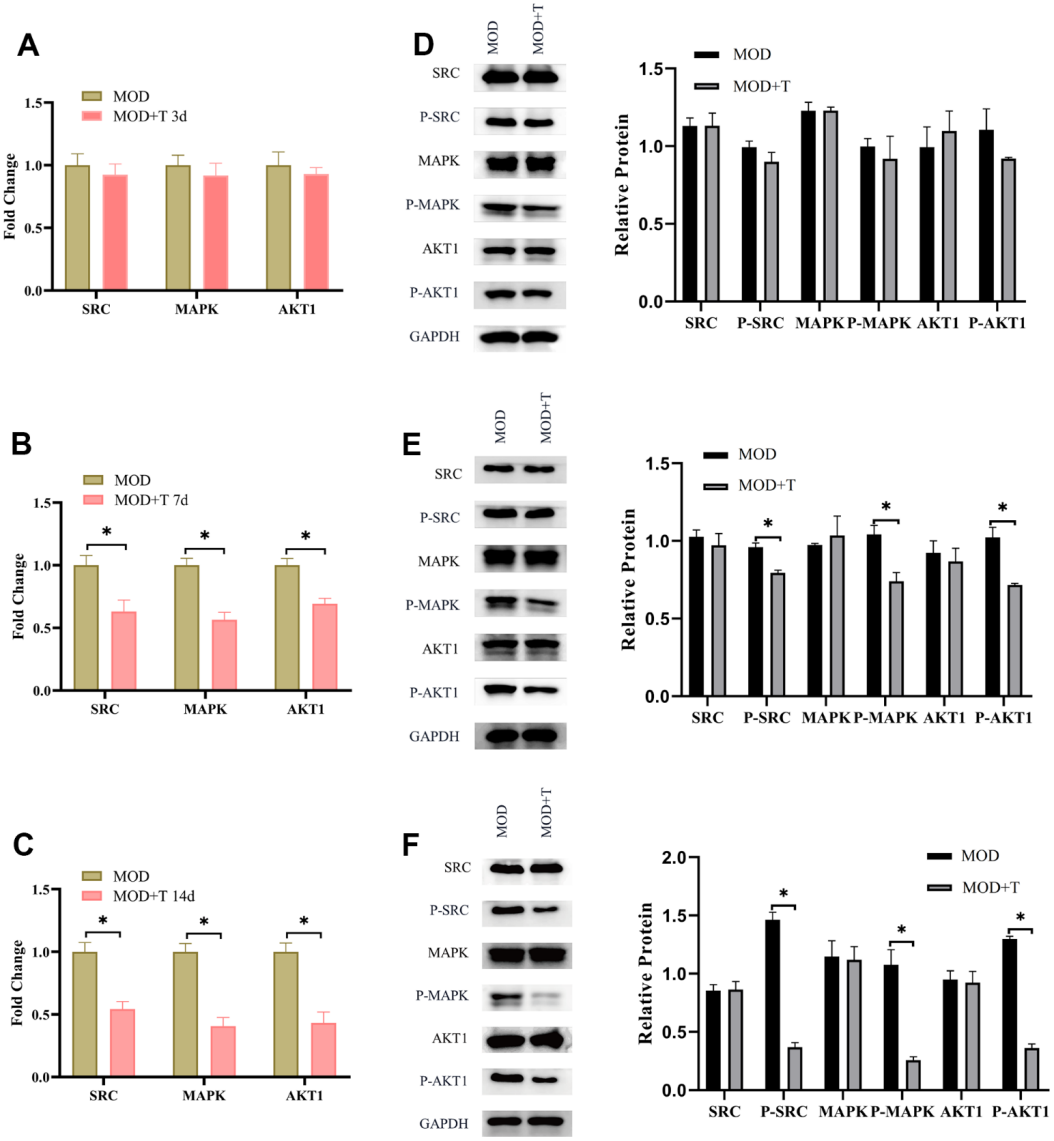
**The TXYF suppresses the phosphorylation of SRC, MAPK and AKT1 in UC.** (**A**–**C**) The mRNA expression level of SRC, MAPK and AKT1 in MOD and MOD+T groups were detected by qRT-PCR at 3^rd^ (**A**), 7^th^ (**B**), and 14^th^ (**C**) day after the UC model was established. (**D**–**F**) The protein expression level of SRC, P-SRC, MAPK, P-MAPK, AKT1, P-AKT1 were detected by WB at 3^rd^ (**D**), 7^th^ (**E**), and 14^th^ (**F**) day after the UC model was established. N=5, *p<0.05.

According to our previous results (Figure 2C), the important targets for the treatment of UC with TXYF include SRC, MAPK, AKT1 in the PPI network. Besides, molecular docking of active ingredients in TXYF revealed that strong binding activity between naringin and MAPK, albiflorin and SRC were observed. In the next step, the underlying molecular mechanism of TXYF was further explored. As shown in Figure 6A–6C, the mRNA expression level of SRC, MAPK and AKT1 was significantly decreased with the treatment of TXYF since 7^th^ day the UC model was established. Furthermore, the phosphorylation of SRC, MAPK and AKT1 was also remarkably reduced since 7^th^ day when the UC model rats were treated with TXYF (*p<0.05). These results indicated that TXYF suppresses the phosphorylation of SRC, MAPK and AKT1 in UC.

## DISCUSSION

Given the unclear pathogenesis, potential progression, and weakened course of UC, the treatment goal for UC has shifted from treating symptoms to mucosal healing [4]. However, traditional treatment methods are mainly limited to controlling inflammation and clinical symptoms, making it difficult to meet the expectations of long-term treatment [20]. Therefore, development of novel therapy is an urgent strategy for UC. The development of new ligands with therapeutic effects on the gut will be a potential pathway for the treatment or prevention of UC [21]. So far, with the widespread use of TCM in different diseases, clinical effects have been confirmed [22]. Here in this study, network pharmacology and bioinformatics were conducted to further understand the etiology of UC and find new potential treatment method.

TXYF has the good clinical application prospects in gastrointestinal diseases [23]. Recently, there is more and more researches paying attention to TXYF. For example, Min Zhang et al. revealed that TXYF alleviates diarrhea-predominant irritable bowel syndrome in rats via the GCN2/PERK-eIF2α-ATF4 signaling pathway [12]. Zhang et al. indicated that TXYF to ameliorate DSS-induced colitis via NF-κB/NLRP3 signaling pathway [16]. These researches suggest the regulation role of TXYF in treating enteritis related diseases. Nevertheless, systems biology research methods such as network pharmacology still need to be used to elucidate mechanisms of TXYF and provide scientific basis for expanding the scope of clinical applications. In addition, we firstly applied analyzed the action targets of TXYF using TCMSP and TCMID databases. The targets of UC were screened in Gene Cards and Online Mendelian Inheritance in Man (OMIM) databases, then the network pharmacology of active ingredient targets was established via Cytoscape.

In this study, the core targets of UC were analyzed. The protective effect of TXYF for UC might be achieved through SRC, MAPK, AKT1, which have a high degree of connectivity in the PPI network. What’s more, our results confirmed that TXYF suppressed the phosphorylation of SRC, MAPK and AKT1 in UC rat model. Some studies have found that AKT can alleviate the symptoms of UC [24], possibly by inhibiting the activation of AKT and downregulating the expression of IL-6, thereby alleviating the inflammatory response in the intestine [25]. Research has also found that SRC-3 can improve DSS induced colitis by enhancing the expression of transcription factor KLF4, which is responsible for the differentiation and maturation of colonic acinar cells, thereby inhibiting inflammation and promoting colonic acinar cell differentiation and maturation [26]. Chen et al. found that inhibition of mRNA expression of apoptosis-related molecules in the MAPK signaling pathway can reduce apoptosis of colonic epithelial cells in colitis mice [27]. In this study, we firstly demonstrated that treatment with TXYF greatly suppressed the protein and mRNA expression levels of SRC, MAPK and AKT1, which might be the potential function mechanism.

KEGG enrichment analysis showed that the TXYF mainly exerts its effect on UC through pathways such as IL-17, TNF-α, HIF-1, etc. Our results verified that TXYF inhibit the expression of HIF-1, IL-17 and TNF-α in UC. IL-17 is a cytokine with strong pro-inflammatory activity [28]. Studies have found an increase in IL-17 expression in the mucosa and serum of patients with colitis, suggesting that IL-17 expression may be related to changes in intestinal mucosal immunity and inflammatory response [29]. The transcription regulatory HIF-1 plays an important role in adaptive hypoxic response [30]. Preliminary studies on mouse colitis based on haptens have shown widespread mucosal hypoxia and accompanying HIF-1 activation during colitis [31, 32]. *Saccharomyces boulardii* improve UC carcinogenesis in mice by reducing TNF-α levels and functions and by rebalancing intestinal microbiota [33]. Anti-TNF-α drugs are used clinically to treat UC [34]. These evidences confirmed that IL-17, TNF, HIF-1signaling pathways are vital in TXYF treating the UC. We also observed these facts that the levels of IL-17, TNF-α, HIF-1 were remarkably inhibited by TXYF (Figure 5D) after 7 and 14 days.

GO biological process enrichment analysis revealed that TXYF in the treatment of UC may be related to biological processes such as endotoxin response, regulation of small molecule metabolism, regulation of reactive oxygen species metabolism, regulation of apoptosis signaling pathways, and negative regulation of cell proliferation. Excessive activation of oxidative stress (OS) can cause excessive production of free radicals, leading to lipid and protein peroxidation, which in turn leads to damage to the intestinal mucosal protective barrier and leads to the onset of UC [35, 36]. Our molecular docking results showed that the binding energies of naringin and MAPK, as well as *albiflorin* and SRC, were -8.5 and -6.4 (kcal/mol), respectively, both less than -5.0, indicating strong binding activity between the drug and the target.

Collectively, TXYF may act on key targets (e.g. SRC, MAPK, AKT1) through active ingredients such as *naringin*, and *abiflorin*, regulating IL-17, TNF, HIF-1 signaling pathways, participating in biological processes such as antioxidant, anti-inflammatory, mucosal barrier improvement, and immune regulation, thus exerting therapeutic effects in UC. The research results are consistent with existing research reports. However, its scientific and practical nature still needs to be analyzed and verified through more experiments. This study provides a scientific basis for the clinical application and research and development of TXYF in treating UC.

## MATERIALS AND METHODS

### Screening for the targets of TXYF

The active chemical components of TCM in the TXYF (*Atractylodes macrocephala*, *Paeonia lactiflora*, *Tangerine peel*, and *Saposhnikovia divaricata*) were obtained through TCM Systems pharmacology platform Traditional Chinese Medicine Systems Pharmacology Database (TCMSP, https://old.tcmsp-e.com/tcmsp.php) and Traditional Chinese Medicine Integrated Database, (TCMID, http://47.100.169.139/tcmid/). The screening criteria are as follows: oral availability (OB) ≥ 30% and Druglikeness (DL) ≥ 0.18. Thereafter, Potential protein targets were identified through the Swiss target prediction database, with a screening condition of probability>=0.1. The screened protein targets were converted into standardized gene names in the UniProt database.

### Screening for the targets of UC

We searched the Online Mendelian Inheritance in Man (OMIM) and Gene Cards databases using the keyword ‘ulcerative colitis’ to obtain target genes related to UC. Typically, targets with a Score value greater than the median are set as potential targets for the UC. The genes with a score value greater than 5 in the Gene Card were left to merge with the OMIM database for deduplication, the obtained genes were related targets to UC.

### Identification of effective targets

Venn analysis was used to intersect the target of TXYF with the targets of UC by Venn tool in TBtools, the intersection target of the two is obtained as effective targets.

### Construction of the active ingredient-effective target network

The active ingredients and effective target genes of drugs were imported into Cytoscape 3.7.2 software for active ingredient-effective target network construction and visualization analysis. Through topological parameter analysis, the main active ingredients are selected based on the degree value.

### Construction of protein-protein integration network

The potential targets of TXYF that with anti-UC effect was imported into the GeneMANIA (http://genemania.org/) database to obtain the interaction relationships between the targets and obtain indirect targets. Indirect targets are added to the action target library and then imported into the String database. Select Homo sapiens was selected in the String database, with a default score of 0.4 to obtain the target interaction network and save its TSV format. The TSV file was imported into the Cytoscape 3.7.2 software for topology analysis of the interaction network, information on the degree values, betweenness centrality (BC), topological coefficient (TC), and proximity centrality (PC) of nodes were obtained. The top 3 targets with degree values are selected as key target proteins.

### Enrichment analysis of target functions and pathways

R software (https://www.r-project.org/) and its backend database org.Hs.eg.db were carried to obtain the gene ID (entrezID) of potential targets. Then DOSE, clusterProfiler, and pathview packages (Bioconductor) were utilized to perform GO functional enrichment analysis (Biological Process (BP), Cellular Component (CC) Molecular Function (MF)) based on the potential targets, with screening Criteria of p-value< 0.05 and q-value > 0.05. The top 10 enriched items in the form of bar charts and bubble charts were displayed.

### The molecule docking for main active ingredients-target of TXYF

The targets of TXYF on UC were searched in the PDB database and saved as PDB format. The ligands were stored in mol2 format for compounds ranked in the top 2-degree values after topological analysis. Auto Dock Tools-1.5.6 was used to dock the potential targets of TXYF with the main compounds in UC through topological analysis. The more stable the conformation of ligand receptor binding, the greater the likelihood of action. The key target ranked high was selected based on the degree value.

### The rat model of UC

30 SPF male SD rats (200-220 g) were purchased from SiPaiFu (Beijing) Biotechnology Co., Ltd. (license number: SCXK (Beijing) 2019-0010). All the animal experiments were performed with the approval of the Ethics Committee of Affiliated Hospital of Jiangxi University of Traditional Chinese Medicine. A disposable rectal administration catheter was used to introduce rats into the colon about 8 cm away from the anus. Each group of rats was slowly injected with 2% TNBS (preparation of 2% TNBS solution: 2ml of 5% TNBS solution+2ml of anhydrous ethanol+1ml of physiological saline, vortex mixing, to obtain 2% TNBS solution), with a dose of 100 mg/kg. When introducing the drug solution, the rats were placed in a position with their heads low and their tails high to prevent the infusion fluid from overflowing and were lifted upside down for 1 min. After modeling, the mice were placed back in their cages and naturally woke up. The SD rats were grouped as follows: UC model group (UC) and UC+TXYF group (UC+TXYF). The UC and UC+TXYF groups were divided into three sub-groups according to the treatment time of TXYF: 3, 7 and 14 days (n=5). After induction of UC, the rats in the group model were treated with normal saline (4 g/kg/d) through gavage administration every two days. The rats in the group UC+TXYF were treated with TXYF (4 g/kg/d) through gavage administration every two days. 3, 7, and 14 days after UC induction, rats were sacrificed, and tissues and blood were collected for experiments. Colon tissue and serum were taken from 5 rats in each group, and the length of the colon was measured. The collected sample were stored at -80° C for further experiments. The dose of TXYF was determined according to our pre-experiment and published references [16, 18].

### The detection of myeloperoxidase (MPO)

The rat colon tissue was prepared into 5% tissue homogenate. The MPO in rat colon tissue was detected by the MPO test kit (#A044-1-1, Nanjing Jiancheng Bioengineering Institute, China) according to the instructions.

### Hematoxylin and eosin (HE) staining

Rat colon tissue was placed in 4% paraformaldehyde for internal fixation for 24h. Then the samples were subjected to gradient dehydration: 70%, 80%, 90%, 95%, ethanol solution for 30 mins, respectively; Anhydrous ethanol, xylene, and paraffin each for 60 mins, respectively. After dehydration, the colon tissues were embedded in paraffin, then cut into tissue slices with a thickness of 5um. Slices were dewaxed: 20 minutes for xylene, 10 minutes for anhydrous ethanol, 5 minutes for 95% ethanol, 5 minutes for 85% ethanol, 5 minutes for 70% ethanol, 5 minutes for pure water. Slices stained: hematoxylin (#C0107, Beyotime, China) for 4 minutes, rinsing with tap water for 10 minutes, differentiation with hydrochloric acid ethanol for 3 s, rinsing with tap water for 10 minutes (returning to blue), eosin (#C0109, Beyotime, China) for 1 minute. Then, anhydrous ethanol was used to dehydrate the slices, xylene was used to permeate the slices, and neutral resin was used for sealing.

### The ELISA detection of HIF-1, IL-17, and TNF-α

The residual blood was removed from the colon tissue of rats. Colon tissue was weighed, cut into small pieces, and thoroughly grounded onto ice to form a homogenate. Thereafter, the homogenate was placed at 5000 × centrifuge for 10 mins and the supernatant was taken for detection. The following test kits were used for detection according to the instructions: Interleukin-17 (IL-17) test kit (#MM-0088R1, Mlbio, USA); tumor necrosis factor-α (TNF-α) Detection kit (#MM-0180R1, Mlbio); hypoxia inducible factor-1 (HIF-1) detection kit (#MM-70607R1, Mlbio).

### QRT-PCR

Total RNA in colon tissue of rats was extracted with RNAiso Plus (#9109, TaKaRa, Japan). The concentration and purity of RNA were determined with NanoDrop 2000C. RNA was reverse transcripted into cDNA using NovoScript® Plus All-in-one 1st Strand cDNA Synthesis SuperMix kit (#E047-01A, Novoprotein, China). Then qRT-PCR reaction was conducted by NovoStart® SYBR qPCR SuperMix Plus (#E096-01B, Novoprotein) according to the following settings: 95° C 35s, 60° C 30s, 95° C 10s, 65° C 5s. After the reaction, amplification curve and fusion curve were confirmed, and the 2^-∆∆CT^ value to represent the relative expression of mRNA were calculated. The sequencing synthesized by Fuzhou Shangya Biosynthesis is as follow: GAPDH-Forward: 5’- ATGGGGAAGGTGAAGGTCG -3’, GAPDH-Reverse: 5’- TCGGGGTCATTGATGGCAACAATA -3’; SRC-Forward: 5’- GTGAGGGAGAGTGAGACCACA -3’, SRC-Reverse: 5’- CGGGAGGTGATGTAGTTTCC -3’; MAPK-Forward: 5’- TTGGACTCGGATAAGAGGATCAC -3’, MAPK-Reverse: 5’- TAGGTCAGGCTCTTCCATTCG -3’; AKT1-Forward: 5’- GGAAGGTGATCCTGGTGAAG -3’, AKT1-Reverse: 5’- CGGTTCTCAGTAAGCGTGTG -3’.

### Western blot

The RIPA buffer (1% Nonidet P40, 0.1% SDS, 0.5% deoxycholate, 50mM Tris (PH7.4) Protease Inhibitor Cocktail) for was used to extract proteins from colon tissue 15 mins. BSA standards were used to produce protein quantitative standard curves. The protein sample was placed in a boiling water bath for 6-10 mins for denaturation. 6%-8% SDS-PAGE gel was configured for protein electrophoresis. Then, membrane was rotated and immersed in the sealing liquid, and it was slowly sealed with a shaking table at room temperature for 1-2 h. Then the protein was transferred to PVDF membrane. The membrane was then cleaned twice by TBST. The membrane was incubated with diluted primary antibody and shaken overnight at 4° C. The first antibodies used were as follows: GAPDH (#60004-1-Ig, 1:8000, Proteintech, China), SRC (ab133283, 1:1000, Abcam, UK), P-SRC (#D7F2Q, 1:1000, Cell Signaling, USA), MAPK (#11257-1-AP, 1:2000, Proteintech), P-MAPK (#80031-1-RR, 1:10000, Proteintech), AKT1 (#60203-2-Ig, 1:10000, Proteintech) and P-AKT1 (#66444-1-Ig, 1:10000, Proteintech). The corresponding secondary antibody HRP-conjugated Affinipure Goat Anti-Mouse IgG (H+L) (#SA00001-1, Proteintech) was successively added and incubated in a shaking table for 1-2 h in dark at room temperature. The developer solution was added and then placed in a BIO-RAD for visualization.

### Availability of data and material

The data and material used to support the findings of this study are included within the manuscript and Supplementary Files.
